# Sinusoidal Obstruction Syndrome Post-allogeneic Transplantation: A Complex Multisystem Challenge

**DOI:** 10.7759/cureus.80078

**Published:** 2025-03-05

**Authors:** Tomas Escobar Gil, Sushrruti Varatharaj, Stephanie K Rosenberg, Gicel J Aguilar, David Santistevan, Keith Azevedo, Andres E Mindiola Romero

**Affiliations:** 1 Internal Medicine, University of New Mexico School of Medicine, Albuquerque, USA; 2 Emergency Medicine, University of New Mexico School of Medicine, Albuquerque, USA; 3 Pathology, University of New Mexico School of Medicine, Albuquerque, USA

**Keywords:** allogenic bone marrow transplant, critical care, malignant hematology, oncology, sinusoidal obstruction syndrome, veno occlusive disease

## Abstract

We present the case of a 41-year-old female with chronic myeloid leukemia (CML) in blast crisis who underwent haploidentical allogeneic stem cell transplantation. Her post-transplant course was complicated by neutropenic sepsis and mucositis, followed by the development of sinusoidal obstruction syndrome (SOS) on post-transplant day (POD) 10, evidenced by rising hyperbilirubinemia (peak 22.2 mg/dL) and significant weight gain primarily due to fluid retention. Imaging confirmed hepatosplenomegaly and portal vein dilation, leading to the SOS diagnosis.

Defibrotide was initiated on POD 16, leading to a progressive decline in bilirubin levels (from 22.2 mg/dL to 2.4 mg/dL by POD 22), suggesting a therapeutic response. However, thrombocytopenia and gastrointestinal hemorrhage necessitated dose interruptions. Supportive care included fluid management, albumin infusions, and diuresis, but hepatorenal syndrome developed, requiring continuous renal replacement therapy (CRRT). On POD 27, she developed acute hypoxic respiratory failure, requiring a high-flow nasal cannula and later vasopressor support for worsening hemodynamic instability. Despite intensified critical care measures, including broad-spectrum antimicrobials and transfusion support, her condition deteriorated, leading to progressive multiorgan failure and transition to comfort care on POD 34.

This case highlights the diagnostic and therapeutic challenges of severe post-transplant SOS, emphasizing the need for early intervention, a tailored risk-benefit assessment of defibrotide, and multidisciplinary critical care strategies for high-risk patients.

## Introduction

Sinusoidal obstruction syndrome (SOS), also known as veno-occlusive disease (VOD), is a rare but severe complication of hematopoietic stem cell transplantation (HSCT) caused by endothelial injury within the hepatic sinusoids [1,2]. Clinically, it manifests as sinusoidal congestion, hepatomegaly, and hyperbilirubinemia, typically within three weeks after transplantation [3,4]. Several factors increase the risk of SOS, including myeloablative conditioning regimens, haploidentical donors, and preexisting liver dysfunction [5,6].

The pathophysiology of SOS is multifactorial, involving endothelial injury, activation of coagulation pathways, inflammatory cytokines, and complement system dysregulation [3]. These mechanisms suggest potential targets for adjunct therapies, such as complement inhibitors, anti-inflammatory agents, or endothelial-protective strategies, which warrant further investigation [7,8]. For survivors of SOS, long-term complications, such as chronic liver disease and portal hypertension, remain significant concerns, with consistent follow-up becoming an important factor in the care of these patients after the event [1].

This case highlights the complexity of SOS in a post-transplant setting, emphasizing the challenges in diagnosis, therapeutic decision-making, and multidisciplinary care. Through this case, we aim to illustrate the clinical course, diagnostic criteria, and therapeutic challenges associated with SOS, contributing to a deeper understanding of this serious complication in hematopoietic stem cell transplantation.

## Case presentation

A 41-year-old female with a history of chronic myelogenous leukemia (CML) in the second accelerated phase underwent busulfan and cyclophosphamide conditioning followed by a haploidentical allogeneic peripheral blood stem cell transplant (allo-PBSCT) from her son. Post-transplant, she developed several complications, including neutropenic sepsis, functional small bowel obstruction (SBO), and posterior pharyngeal and esophageal mucositis.

On post-transplant day (POD) 10, she presented with progressive jaundice and significant weight gain of 3 kg (>5% of weight gain), primarily due to fluid retention, as evidenced by edema and ascites on clinical examination. Laboratory studies revealed hyperbilirubinemia (peak total bilirubin 22.2 mg/dL, normal range 0.1-1.2 mg/dL) with normal transaminases and alkaline phosphatase levels, consistent with an isolated hepatic process.

**Figure 1:**
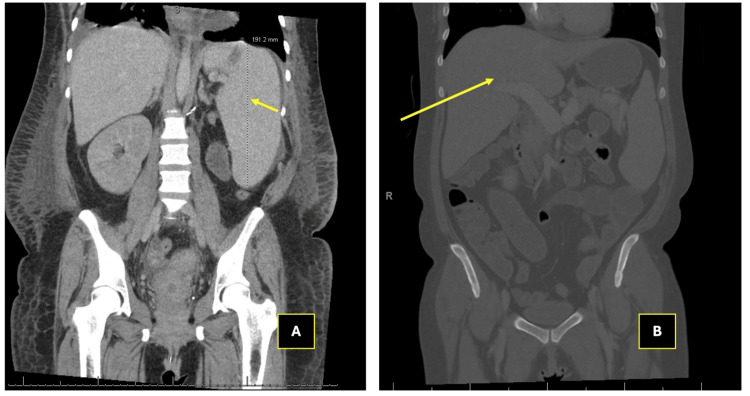
Computed tomography of the abdomen and pelvis with veno-occlusive disease findings A: The yellow arrow points to splenomegaly, evidenced by an enlarged liver measuring up to 19 cm in the craniocaudal dimension. This finding aligns with clinical features of sinusoidal obstruction syndrome (SOS), including sinusoidal congestion and hepatocyte swelling caused by endothelial damage. B: The yellow arrow indicates hepatomegaly with an enlarged liver craniocaudal dimension of 22 cm. The overall architecture reflects the characteristic congestion and enlargement associated with SOS, which is secondary to impaired sinusoidal blood flow.

**Figure 2:**
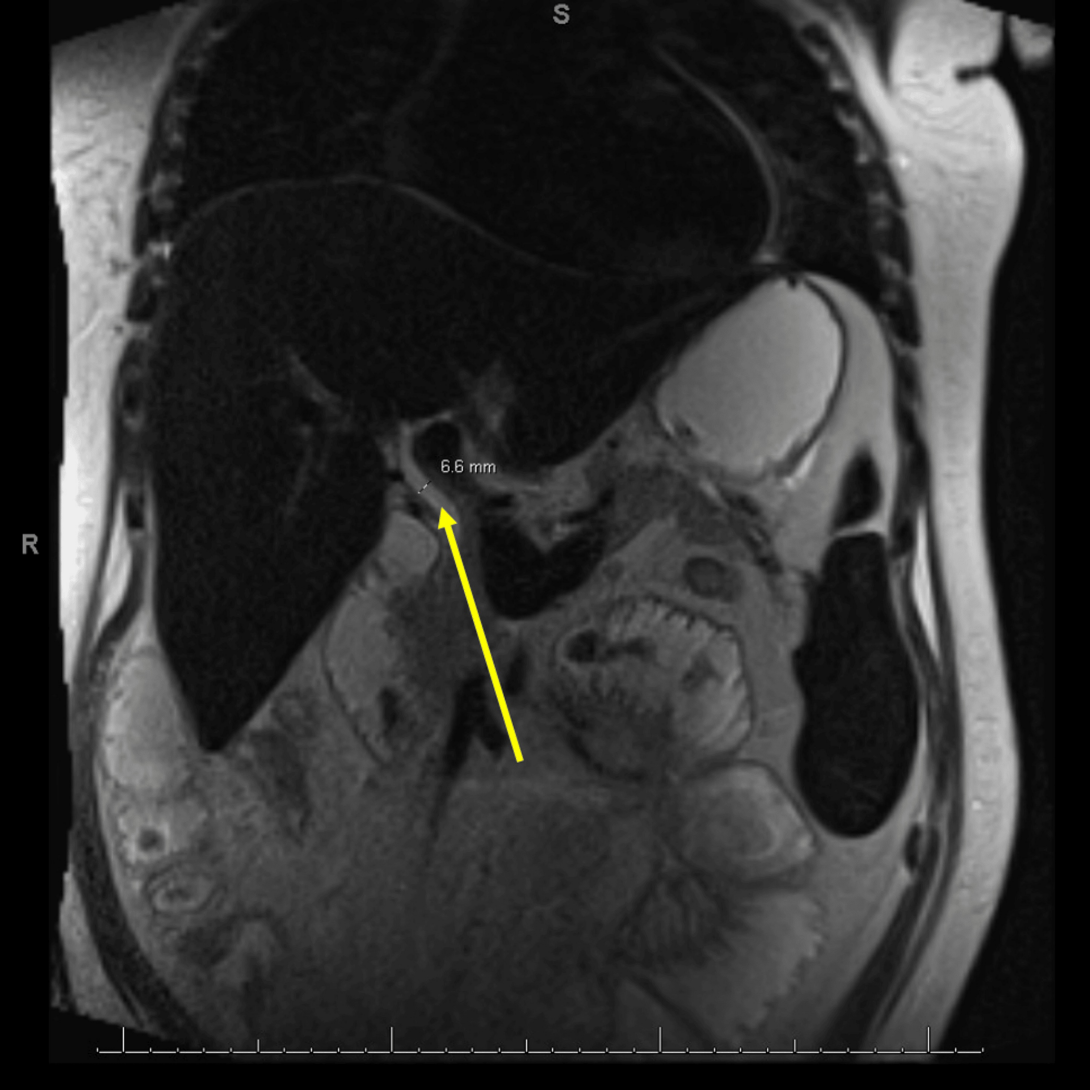
Magnetic resonance cholangiopancreatography with veno-occlusive disease findings Magnetic resonance cholangiopancreatography (MRCP) demonstrates hepatosplenomegaly with diffuse T2 low signal intensity in the liver and spleen, suggesting iron deposition (hemosiderosis). The yellow arrow points to the extrahepatic bile duct, measuring up to 6.6 mm. No intra- or extrahepatic biliary dilation is seen, ruling out obstructive cholestasis. Partially visualized dilated small bowel loops indicate concurrent gastrointestinal involvement, consistent with the patient’s history of small bowel obstruction and enteritis. These findings support the diagnosis of sinusoidal obstruction syndrome (SOS).

**Figure 3:**
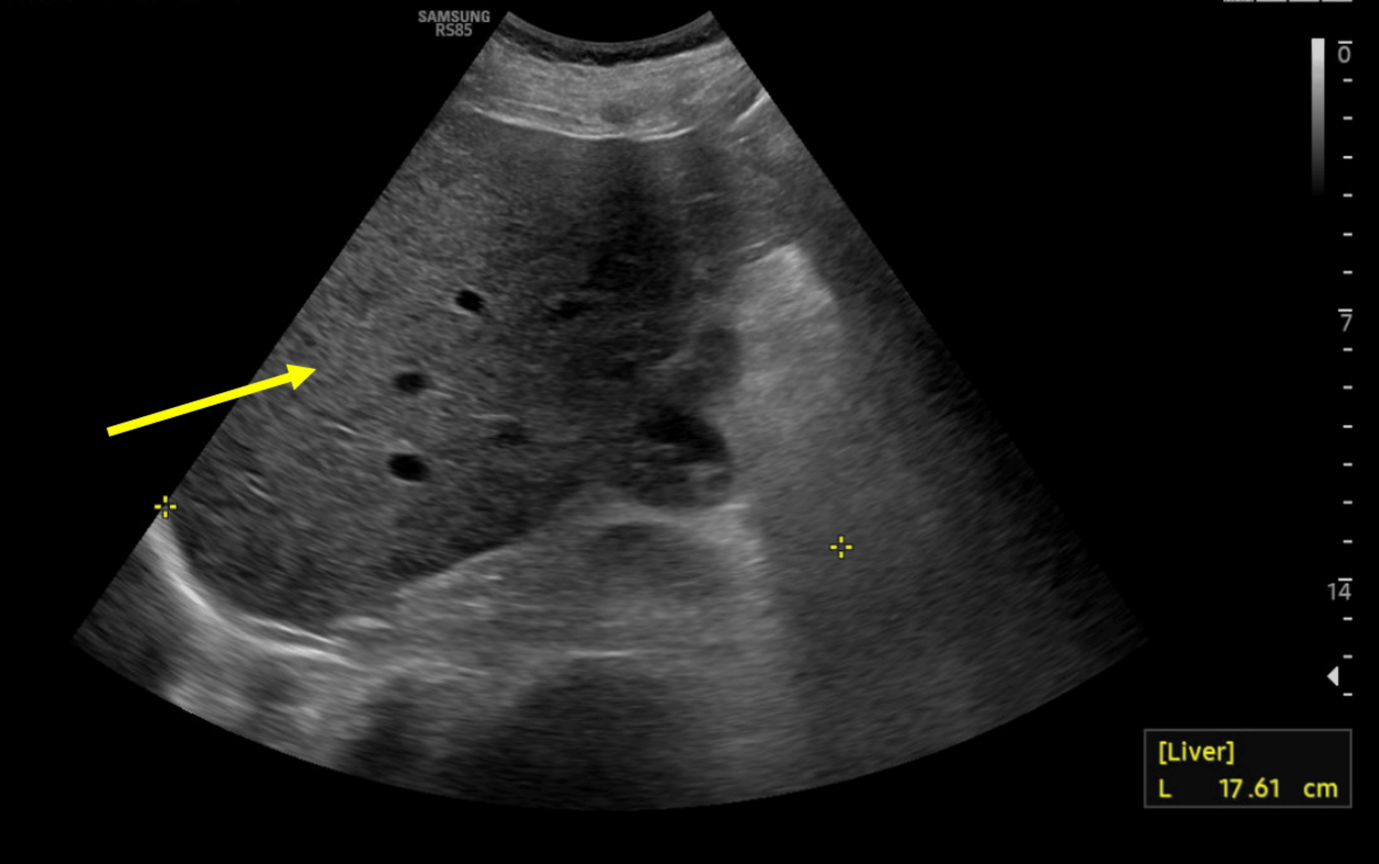
Liver ultrasound of the patient showing hepatomegaly with no biliary dilation Liver ultrasound shows hepatomegaly (right lobe 17.6 cm, see yellow arrow). The main portal vein has normal hepatopetal flow, ruling out portal vein thrombosis. No intrahepatic biliary dilation or ascites is present, supporting sinusoidal obstruction syndrome (SOS) as the cause of liver dysfunction.

Imaging of the abdomen and pelvis, including MRI, CT, and ultrasounds, demonstrated hepatosplenomegaly and portal vein dilation without evidence of biliary obstruction (Figures 1-3). These findings, along with her clinical course, were diagnostic of SOS.

Defibrotide was initiated on POD 16, leading to a progressive decline in bilirubin levels (from 22.2 mg/dL to 2.4 mg/dL by POD 22), suggesting a therapeutic response. However, the concurrent use of supportive measures, including fluid management and diuresis, makes it difficult to determine whether the reduction was solely attributable to defibrotide or multifactorial. Despite these measures, her course was complicated by refractory thrombocytopenia and hematochezia, necessitating periods of defibrotide cessation.

**Figure 4:**
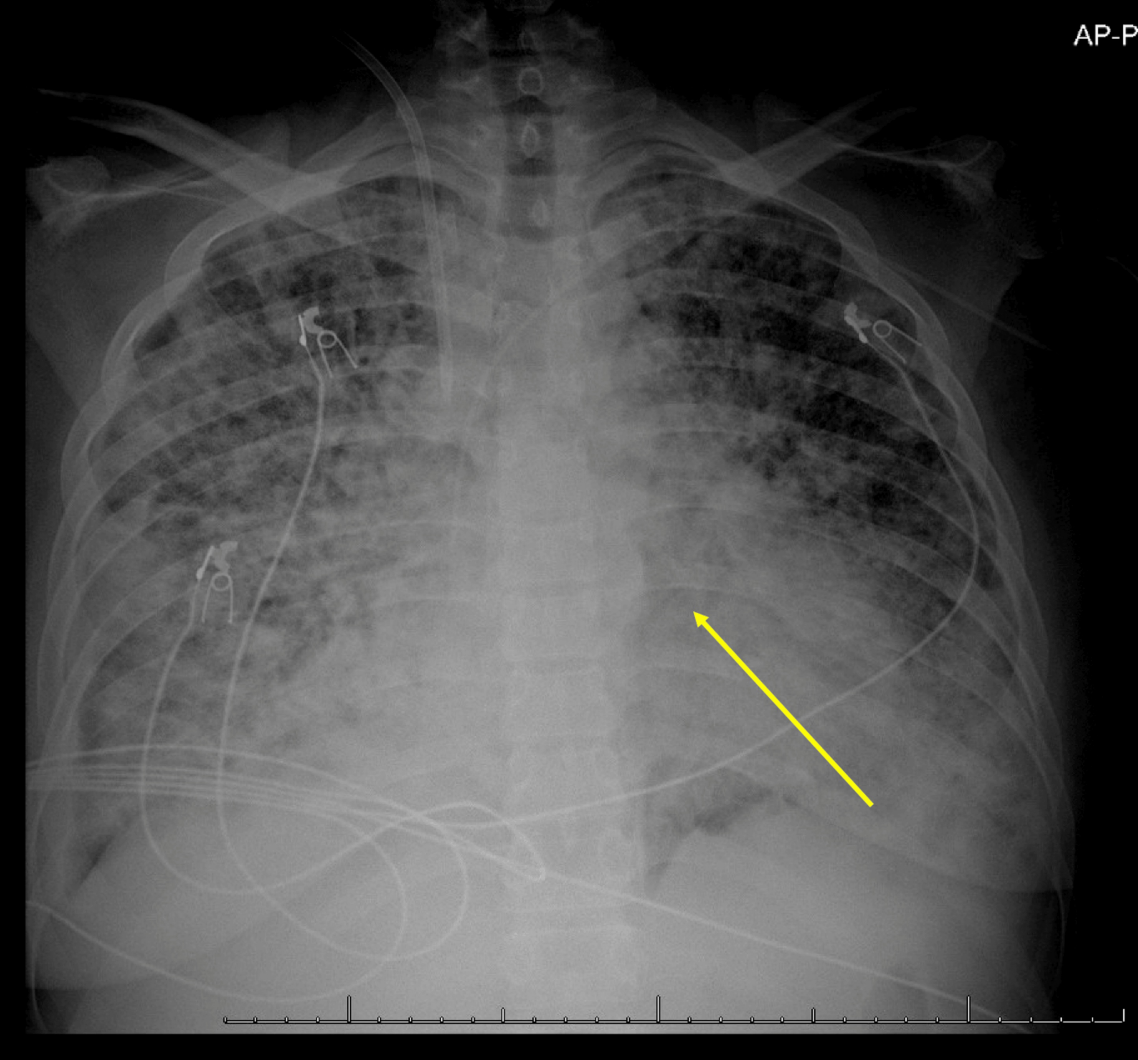
Chest X-ray demonstrating pulmonary edema in sinusoidal obstruction syndrome Anteroposterior (AP) chest X-ray shows diffuse bilateral pulmonary opacities consistent with pulmonary edema (see yellow arrow). Notably, there is increased interstitial marking and alveolar opacities, predominantly in the lower lung fields, suggesting fluid overload. The cardiac silhouette is mildly enlarged, and there is bilateral pleural effusion, more pronounced on the right.

On POD 27, she was transferred to the medical intensive care unit (MICU) for acute hypoxic respiratory failure, requiring a high-flow nasal cannula. A chest X-ray showed severe pulmonary edema (Figure 4). Despite diuresis with furosemide, her condition worsened with the development of acute kidney injury, mixed acidosis, and bacteremia (*Klebsiella pneumoniae*, *Stenotrophomonas maltophilia*, and vancomycin-resistant *Enterococcus faecium* were all isolated). Continuous renal replacement therapy (CRRT) was initiated, and vasopressors were required to maintain hemodynamic stability. The patient's critical condition progressed to severe multisystem dysfunction, with marked anemia, thrombocytopenia, hepatic injury, coagulopathy, and acute kidney injury (Table 1). Increasing bilirubin and hypoalbuminemia without hepatic obstruction further suggested SOS while the marked hyperferritinemia evidenced her systemic inflammation.

**Table 1:** Laboratory values This table provides an overview of the patient's critical lab values, showing marked abnormalities, including severe anemia, thrombocytopenia, hyperferritinemia, hyperbilirubinemia, metabolic acidosis, and kidney dysfunction. Abbreviations: L (low), N (normal), H (high), MCV (mean corpuscular volume), MCHC (mean corpuscular hemoglobin concentration), NRBC (nucleated red blood cell), INR (international normalized ratio), APTT (activated partial thromboplastin time), AST (aspartate aminotransferase), SGOT (serum glutamic-oxaloacetic transaminase), ALT (alanine aminotransferase), SGPT (serum glutamic pyruvic transaminase), GGT (gamma-glutamyl transferase)

Category	Laboratory Test	Patient Value	Reference Range	Flag
Complete Blood Count	RBC	2.06 million/µL	4.2 - 5.4 million/µL	L
	HGB	5.9 g/dL	12.0 - 15.5 g/dL	L
	Hematocrit	19%	37 - 47 %	L
	MCV	89 fL	80 - 100 fL	N
	MCHC	31.4 g/dL	32 - 36 g/dL	L
	Platelet Count	7 thousand/µL	150 - 450 thousand/µL	L
	Neutrophil Count	6 thousand/µL	1.8 - 7.7 thousand/µL	N
	Lymphocyte Count	0 thousand/µL	1.0 - 3.0 thousand/µL	L
	Monocyte Count	0.1 thousand/µL	0.2 - 1.0 thousand/µL	N
	Eosinophil Count	0 thousand/µL	0.0 - 0.5 thousand/µL	N
	Basophil Count	0 thousand/µL	0.0 - 0.2 thousand/µL	N
	NRBCs	0%	0.0 - 0.5 %	N
Coagulation Panel	Prothrombin Time	13.4 sec	10 - 13 sec	H
	INR	1.19	0.8 - 1.1	H
	APTT	31.4 sec	25 - 35 sec	H
	Fibrinogen	716 mg/dL	200 - 400 mg/dL	H
Electrolytes	Sodium	138 mEq/L	135 - 145 mEq/L	N
	Potassium	4.2 mEq/L	3.5 - 5.0 mEq/L	N
	Chloride	103 mEq/L	98 - 106 mEq/L	N
	Bicarbonate	17 mEq/L	22 - 28 mEq/L	L
	Creatinine	1.55 mg/dL	0.6 - 1.2 mg/dL	H
Liver Function Tests	Total Protein	5.8 g/dL	6.0 - 8.3 g/dL	L
	Albumin	2.2 g/dL	3.5 - 5.0 g/dL	L
	Globulin	3.6 g/dL	2.0 - 3.5 g/dL	N
	AST (SGOT)	52 U/L	0 - 40 U/L	H
	ALT (SGPT)	19 U/L	7 - 56 U/L	N
	Alkaline Phosphatase	240 U/L	44 - 147 U/L	H
	Bilirubin, Total	22 mg/dL	0.1 - 1.2 mg/dL	H
	Bilirubin, Direct	21.3 mg/dL	0.0 - 0.3 mg/dL	H
	Bilirubin, Indirect	0.7 mg/dL	0.2 - 0.9 mg/dL	N
	GGT	55 U/L	9 - 48 U/L	H
	Ammonia	82 µmol/L	15 - 45 µmol/L	H
Other Tests	Ferritin	38,850 ng/mL	20 - 300 ng/mL	H

After extensive discussions with her family, her care was transitioned to comfort measures. She expired on POD 34.

## Discussion

**Figure 5:**
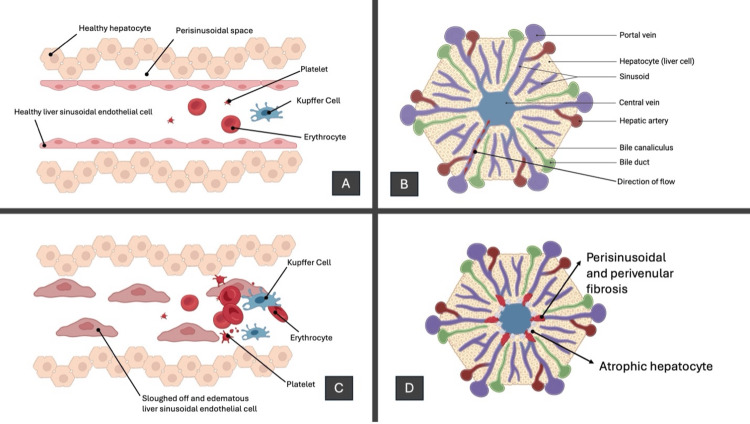
Veno-occlusive syndrome pathophysiology Panel A: Normal hepatic sinusoid with intact endothelial cells, Kupffer cells, and healthy hepatocytes. Panel B: Blood flows from the portal vein and hepatic artery through sinusoids to the central vein while bile flows in the opposite direction through bile canaliculi to the bile duct. Panel C: Endothelial injury causes cell sloughing, microthrombi formation, and obstruction of sinusoids, leading to congestion and hepatocellular injury. Panel D: Progressive damage results in perisinusoidal fibrosis, hepatocyte atrophy, and portal hypertension, causing irreversible liver dysfunction. This illustration is the authors' own creation.

Endothelial injury, activation of coagulation pathways, inflammatory cytokine release, and complement system dysregulation all contribute to the inflammation in SOS. The initial endothelial damage leads to the loss of sinusoidal integrity, promoting microthrombi formation and sinusoidal congestion. This cascade triggers an exaggerated inflammatory response, further amplifying endothelial dysfunction by releasing cytokines such as tumor necrosis factor-alpha (TNF-α) and interleukins. Concurrently, dysregulation of the complement system exacerbates vascular injury and promotes fibrin deposition, ultimately resulting in hepatocellular damage, portal hypertension, and multiorgan dysfunction (Figure 5) [3].

SOS is diagnosed using well-established criteria, including the Modified Seattle Criteria, Baltimore Criteria, and the European Society for Blood and Marrow Transplantation (EBMT) criteria for Adults (Table 2) [2,3]. The Seattle Criteria focus on the presence of hyperbilirubinemia >2 mg/dL, hepatomegaly or right upper quadrant pain, and weight gain due to fluid retention [2]. The Baltimore Criteria emphasize bilirubin elevation >2 mg/dL accompanied by at least two additional signs: hepatomegaly, ascites, or weight gain >5% [2]. The EBMT criteria provide a more refined framework, requiring classical features of SOS such as hyperbilirubinemia, weight gain, painful hepatomegaly, and ascites within 21 days post-transplant [3]. These criteria also extend to severe SOS, defined by multiorgan dysfunction (e.g., renal or respiratory failure) [2].

**Table 2:** Comparison of diagnostic criteria for sinusoidal obstruction syndrome (SOS) European Society for Blood and Marrow Transplantation (EBMT)

Criteria	Timeframe	Bilirubin Threshold	Other Diagnostic Features	Severity Classification
Modified Seattle	Within 21 days post-transplant	≥2 mg/dL	At least 2 of the following: hepatomegaly, right upper quadrant pain, weight gain (>5%)	No formal severity grading
Baltimore	Within 21 days post-transplant	≥2 mg/dL	At least 2 of the following: hepatomegaly, ascites, weight gain (>5%)	No formal severity grading
EBMT	Anytime post-transplant	Not required	Requires all 4: bilirubin elevation, weight gain, painful hepatomegaly, and ascites	Yes (mild, moderate, severe, very severe)

Our patient fulfilled all of these criteria. She presented with hyperbilirubinemia peaking at 22 mg/dL (normal range 0.1-1.2 mg/dL), significant weight gain exceeding 5%, hepatosplenomegaly evident on imaging, and clinical ascites. Additionally, her rapid progression to multiorgan dysfunction, including acute kidney injury and respiratory failure, aligns with the EBMT classification of severe SOS. This alignment underscores the utility of these diagnostic tools in the early recognition and stratification of disease severity, which is critical for timely intervention with therapies such as defibrotide, the therapy used for this patient.

This medication was discovered in the 1970s, and it is a polydisperse oligonucleotide from porcine DNA initially studied for antithrombotic effects. Its endothelial-protective properties were later identified, leading to its use in sinusoidal obstruction syndrome. It was approved for SOS in Europe in 2013 and in the U.S. in 2016. It stabilizes endothelial cells, reduces inflammation, and enhances fibrinolysis without systemic anticoagulation [7]. Administered at 6.25 mg/kg intravenously every 6 hours for a minimum of 21 days, defibrotide has been shown to improve survival by restoring sinusoidal blood flow and mitigating vascular damage. However, its use is not without risk, especially in thrombocytopenic patients where bleeding is a significant concern [7].

While defibrotide resulted in an early reduction in bilirubin levels in this patient, her clinical course was complicated by thrombocytopenia and gastrointestinal bleeding, requiring interruptions in therapy. These complications, along with her respiratory failure, acute kidney injury, and recurrent infections, highlight the challenges of managing SOS in patients with multiple comorbidities.

Given the role of complement activation in endothelial damage, complement inhibitors like eculizumab have been explored in transplant-associated thrombotic microangiopathy (TA-TMA) and may offer potential for SOS treatment [8]. This patient’s rapid deterioration despite defibrotide therapy highlights the need for additional therapeutic strategies, such as complement blockade or anti-inflammatory agents, to mitigate endothelial injury.

Prognostic tools, such as the model developed by Beorman et al., incorporating variables such as bilirubin levels and weight gain could help predict outcomes and guide clinical decisions [9]. Preventive strategies, including continuous low-dose heparin infusions, have shown some potential to reduce the incidence of SOS, but their role in adults remains uncertain and requires further investigation [10].

Ultimately, our case highlights the importance of recognizing SOS early and balancing the risks and benefits of therapies like defibrotide. Multidisciplinary collaboration is crucial in managing post-transplant complications, and ongoing research is needed to refine both diagnostic tools and treatment strategies for this life-threatening condition.

## Conclusions

This case highlights the severe and multifaceted nature of sinusoidal obstruction syndrome (SOS) following allogeneic hematopoietic stem cell transplantation. Despite early recognition and initiation of defibrotide therapy, the patient's clinical course was complicated by refractory thrombocytopenia, hemorrhagic events, and progressive multiorgan dysfunction, ultimately leading to a poor outcome. The interplay between endothelial injury, coagulation dysregulation, and inflammatory pathways underscores the complexity of SOS pathophysiology and the challenges in its management.

Future research should focus on refining risk stratification models by incorporating biomarkers of endothelial injury and inflammatory response, as well as exploring novel therapeutic strategies, such as complement inhibitors, endothelial-protective agents, and targeted anti-inflammatory therapies, to mitigate endothelial damage and improve survival in patients with severe SOS.
